# N-Terminal Pro-Brain Natriuretic Peptide Predicts Long-Term Technique Failure in Patients Undergoing Peritoneal Dialysis

**DOI:** 10.3390/jcm7120557

**Published:** 2018-12-16

**Authors:** Chia-Ter Chao, Chih-Kang Chiang, Jenq-Wen Huang, Kuan-Yu Hung

**Affiliations:** 1 Department of Medicine, National Taiwan University Hospital BeiHu Branch, College of Medicine, National Taiwan University, Taipei 108, Taiwan; b88401084@gmail.com; 2 Department of Integrative Diagnostics and Therapeutics, National Taiwan University Hospital, Taipei 100, Taiwan; ckchiang@ntu.edu.tw; 3 Nephrology Division, Department of Internal Medicine, National Taiwan University Hospital, Taipei 100, Taiwan; kyhung@ntu.edu.tw; 4 Department of Internal Medicine, National Taiwan University Hospital Hsinchu Branch, Hsinchu City 300, Taiwan

**Keywords:** brain natriuretic peptide, end-stage renal disease, peritoneal dialysis, mortality, technique failure

## Abstract

It is unclear whether N-terminal pro-brain type natriuretic peptide (NT-proBNP) level can be a biomarker for technique failure among long-term peritoneal dialysis (PD) patients. We prospectively included end-stage renal disease patients undergoing PD from a single center between December 2011 and December 2017. We divided the cohort into high or low NT-proBNP groups and analyzed the risk factors associated with the incidence of technique failure using Cox proportional hazard regression analysis. A total of 258 chronic PD patients (serum NT-proBNP, 582 ± 1216 ng/mL) were included. After a mean follow-up of 3.6 years, 49.6% of PD patients developed technique failure and switched to hemodialysis, while 15.5% died. Cox proportional hazard regression analyses accounting for age, gender, diabetes, renal clearance, C-reactive protein, and hydration status, showed that higher natural log transformed NT-proBNP levels (hazard ratio [HR] 1.13, *p* < 0.01) were predictive of an increased risk of technique failure, and were also predictive of an increased risk of mortality (HR 1.56, *p* < 0.01). Consequently, NT-proBNP might be an under-recognized biomarker for estimating the risk of technique failure, and regular monitoring NT-proBNP levels among PD patients may assist in their care.

## 1. Introduction

The population of patients with chronic kidney disease (CKD) and end-stage renal disease (ESRD) is increasing globally, along with the rising trend of aging population, rising incidence of hypertension, diabetes mellitus (DM), and acute kidney injury (AKI) [1,2,3]. Among the available therapeutic modalities for ESRD, peritoneal dialysis (PD) has been found to demonstrate an early survival advantage for these patients compared to hemodialysis, conferring a better quality of life and entailing a significantly lower healthcare cost [4,5]. Despite these perceived benefits, PD is still under-utilized and less promoted in most developed countries, and it has been found that the proportion of ESRD patients receiving PD has gradually declined over time in Japan and several European countries [4]. Factors contributing to this phenomenon may include discrepancies in the reimbursement policy, physicians’ perceptions of a better hemodynamic status associated with hemodialysis, staff availability, familiarity with certain modality, and most important of all, the risk of PD technique failure necessitating a switch to hemodialysis [6]. Technique failure among chronic PD patients is frequently accompanied by the placement of temporary vascular access, forced adaptation of their lifestyle to twice or thrice weekly hemodialysis, unplanned hospitalizations, and an increase in the cost [7]; consequently, the quest for identifying important and modifiable risk factors for technique failure assumes importance.

Previous reports have shed light on factors that could affect the incidence of technique failure in PD patients, including center-specific and patient related factors [8]. Those receiving management in a larger PD service center have a lower incidence of technique failure, while the presence of an advanced age, DM, high or high average peritoneal equilibration test (PET) status, and an increased dialysate glucose load are associated with a higher risk [8,9,10,11]. However, few existing studies focused on the laboratory parameters to examine the risk of technique failure, and those which included such variables mainly focused on hemoglobin, serum albumin, calcium/phosphate, or C-reactive protein (CRP) levels [8,10,12]. It is suggested that fluid overload increases the risk of technique failure among chronic PD patients [13], but whether the volume-sensitive laboratory parameters play a role in modifying the risk of technique failure is still unclear.

N-terminal pro-brain natriuretic peptide (NT-proBNP) is the inactive form of one of the natriuretic peptide family members, BNP, produced by the ventricular myocytes and upregulated during cardiac failure or myocardial infarction. NT-proBNP has been shown to predict the risk of arterial stiffness, volume overload, and cardiovascular mortality in chronic PD patients [14,15,16] but whether NT-proBNP level can be a biomarker for subsequent technique failure is rarely addressed. Moreover, not all factors that impair patient survival exhibit similar influence on the incidence of technique failure among chronic PD patients [12]. It can be unwise to presume that factors correlating with a worse outcome of PD patients act similarly with regard to technique failure. We hypothesized that high NT-proBNP levels could predict the risk of technique failure in chronic PD patients, independent of other clinical and fluid status related variables. In this study, we prospectively studied a PD cohort to examine this issue.

## 2. Methods

### 2.1. Ethical Approval

The protocol was approved by the meeting of Research Ethics Committee of the National Taiwan University Hospital (NTUH-REC 200906084RPC), and the procedure adhered to the Declaration of Helsinki. All participants provided written informed consent for study participation.

### 2.2. Establishment of the Index PD Cohort

Patients with ESRD initiated on long-term PD were prospectively included from the PD center of National Taiwan University Hospital between December 2011 and December 2017. Initiation of PD was based on the feasibility of using PD as renal replacement therapy for more than three months. All patients received a Tenckhoff catheter implantation with glucose, icodextrin, or amino acid-based dialysate as necessary, to achieve optimal azotemic management and ultrafiltration. Continuous ambulatory PD or automated PD was provided based on the concurrence between clinicians and patients. The inclusion criteria consisted of age ≥ 20 years, having ESRD and receiving PD for more than three months regardless of whether PD was their first treatment modality, and the ability to communicate with research staff. Exclusion criteria comprised refusal to participate in the study during the interview and inadequate data available for analysis.

After enrollment, demographic data (age, gender, and body mass index (BMI)), the duration of PD, the etiology of ESRD, comorbidities, medications with outcome influences, and vital parameters (systolic and diastolic blood pressure (BP)) were obtained. The blood tests included hemogram, renal function assay (urea nitrogen and creatinine), nutritional parameter (albumin), parathyroid hormone, and inflammatory parameter (C-reactive protein (CRP)). Part of the serum sample was cryo-preserved-at −20 °C for subsequent analysis. In addition, participants underwent body composition analysis using a bioimpedance-based instrument (BCM; Fresenius Medical Care, a Deutschland GmbH, Bad Homburg, Germany), which is widely used in ESRD patients, with credible results compared to dual energy X-ray absorptiometry [17,18]. Cardiothoracic ratio (CTR) was calculated from the plain chest film which was routinely taken annually for all ESRD patients in Taiwan.

Sera were tested for NT-proBNP levels using an ELISA Kit for Human NT-proBNP (ElAab Company, China). The range of detection for this assay is between 0.312 and 20 (ng/mL). For data outside the kit-specified detection range, samples were diluted 90–210 folds for measurement. The intra and inter assay coefficients of variability were less than 5.9% and 7.8%, respectively.

### 2.3. Endpoints

Participants was prospectively followed up since the day they first underwent body composition analysis, until death, receiving renal transplantation, care transfer to other center, technique failure with modality switch to hemodialysis, or September 30th, 2018, whichever was earliest. The primary outcome of this study was the finding of technique failure during follow-up, defined as the transition of ESRD therapeutic modality from PD to hemodialysis for ≥1 month [19]. Participants, who switched their treatment modality multiple times, were counted once, based on the date of their first modality switch. The secondary outcome was mortality during follow-up.

### 2.4. Statistical Analysis

Continuous and categorical variables were expressed as mean ± standard deviation (SD) and numbers with percentages, respectively, and compared using the Student’s *t*-test and the Chi-square test, respectively. Poisson analysis was used for comparing peritonitis rate. We first examined the distribution of serum NT-proBNP levels in the entire cohort and created a distribution plot. The median value of NT-proBNP was subsequently selected to stratify the entire cohort into halves (high vs. low NT-proBNP levels). We compared the clinical features including demographic data, the etiology of ESRD, comorbidity, concurrent medications, and physical parameters; laboratory profiles including hemogram, serum biochemistry, NT-proBNP levels, and PD-related characteristics (fourth hour of dialysate glucose to baseline ratio and dialysate to blood creatinine ratio, peritoneal, renal, and total Kt/V); peritonitis rate; and body composition data between those with high and low NT-proBNP levels. We further performed correlation analyses between NT-proBNP levels and variables exhibiting significant differences between them, with high and low NT-proBNP or variables that were deemed important for patient outcome. A multivariate linear regression analysis was performed to examine significant determinants of serum NT-proBNP levels at baseline.

After follow-up, the participants were stratified based on the development of primary or secondary outcomes, and compared with respect to the clinical features outlined above. Kaplan–Meier technique was used to construct survival and technique failure-free curves, and between group comparison was done by a log-rank test. Finally, Cox proportional hazard regression analyses were performed to determine important risk factors for the primary and secondary outcomes, incorporating variables deemed clinically important for outcomes, and serum NT-proBNP levels.

## 3. Results

**Figure 1:**
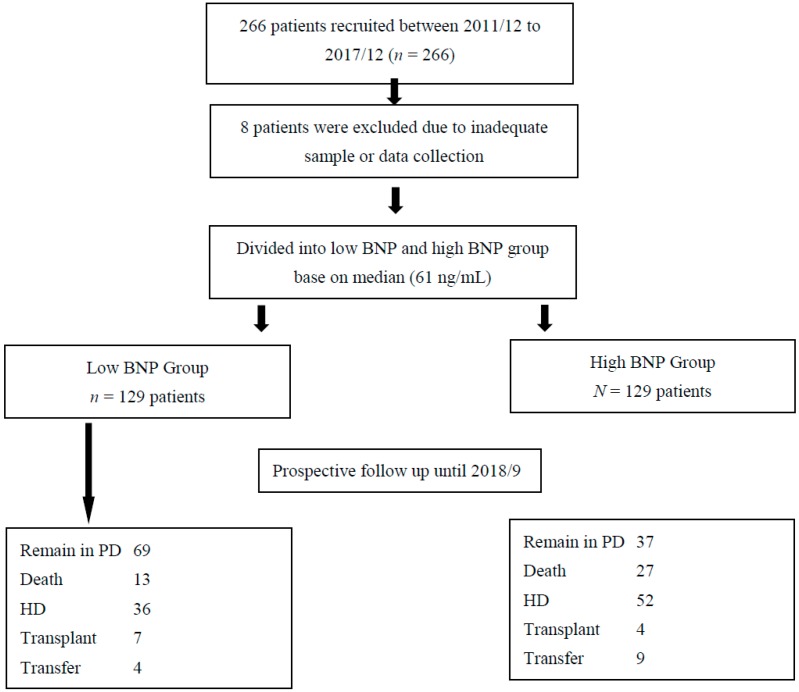
Flow chart of patient selection for this study. BNP, brain natriuretic peptide; HD, hemodialysis; PD, peritoneal dialysis.

**Figure 2:**
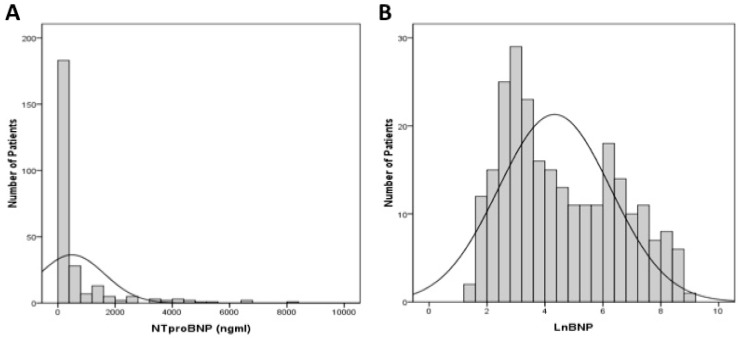
Distribution of serum NT-proBNP in the entire cohort. (**A**) NT-proBNP levels. (**B**) Natural log-transformed NT-proBNP levels. NT-proBNP, N-terminal pro-Brain type natriuretic peptide.

During the study period, we prospectively included 266 ESRD patients undergoing long term PD at our center (Figure 1). After excluding 8 patients with inadequate clinical or laboratory data, 258 long-term PD patients were included and subsequently analyzed. The mean serum NT-proBNP levels were 582 ± 1216 ng/mL, and the distribution of serum NT-proBNP in this cohort is shown in Figure 2A. Some participants had extremely high NT-proBNP levels, and to facilitate analysis, we used the natural log transformed NT-proBNP (LnBNP) levels in the remaining part of analysis (Figure 2B). In addition, we chose the median values (61 ng/mL) of all PD patients in this study as the cut-off point to categorize our PD participants into high and low NT-proBNP groups.

PD patients with high NT-proBNP levels (mean 1141 ± 1530 ng/mL) had a significantly longer duration of PD (*p* < 0.01) prior to enrolment, a higher prevalence of heart failure (*p* < 0.01), higher peritoneal Kt/V (*p* < 0.01) but lower renal Kt/V (*p* < 0.01), and lower Hb (*p* < 0.01) levels than those with low NT-proBNP levels (mean 23 ± 14 ng/mL) (Table 1). Those with high NT-proBNP had significantly higher CTR (*p* < 0.01), extracellular to intracellular water ratio (E/I) (*p* < 0.01), and greater over-hydration (OH) severity (*p* < 0.01), than those with low levels (Table 1). There was no significant difference with regard to PET status between patients with low and high NT-proBNP levels. LnBNP levels correlated significantly with gender (*p* < 0.01), renal Kt/V (*p* < 0.01), Hb (*p* < 0.01) levels, CTRs (*p* < 0.01), and relative OH extent (*p* < 0.01) (Table 2). A multivariate linear regression analysis revealed that lower renal Kt/V (β = −1.55, *p* < 0.01), higher degree of overhydration (β = 0.08, *p* < 0.01), CTR (β = 0.04, *p* = 0.02), and the presence of heart failure (β = 0.97, *p* < 0.01) were independently associated with higher NT-proBNP levels at baseline.

**Figure 3:**
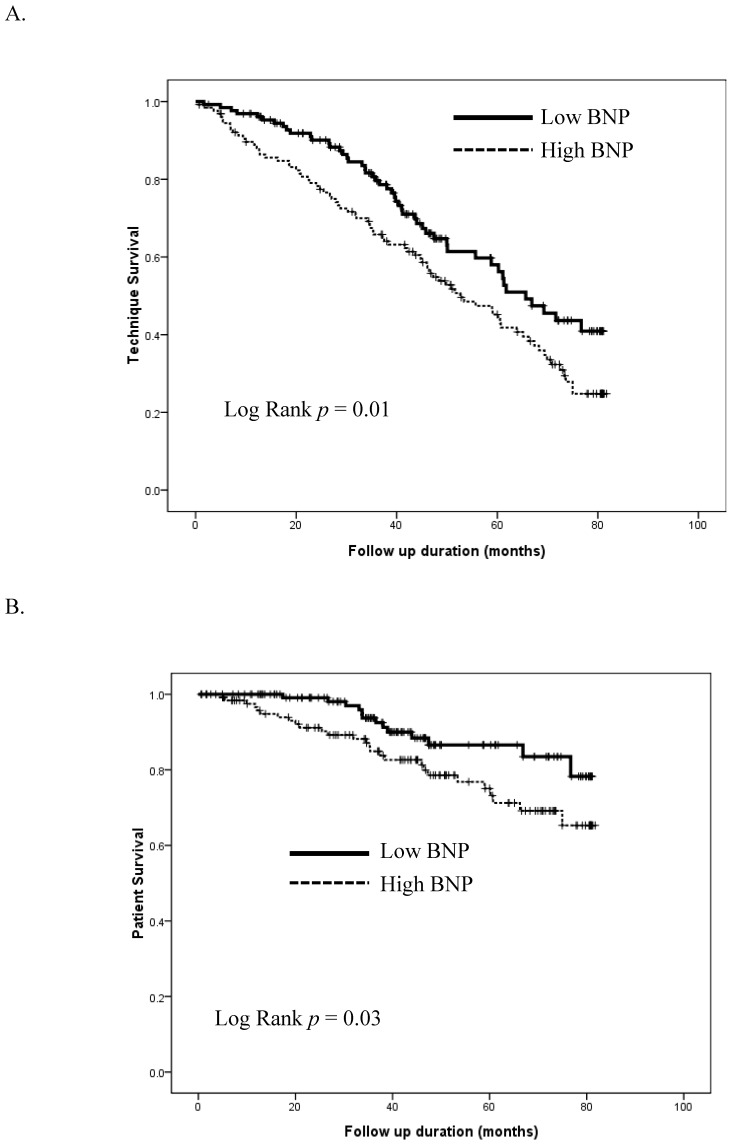
Kaplan–Meier based technique survival curves (**A**) and overall survival curves (**B**) of PD patients with high or low NT-proBNP levels at baseline. BNP, brain-type natriuretic peptide; PD, peritoneal dialysis.

After 3.6 years of follow up, 128 (49.6%) PD patients developed technique failure and switched to hemodialysis, whiled 40 (15.5%) died. The incidence of PD peritonitis during this period was 1.73 episodes per 100 months, without significant differences between those with high and low NT-proBNP levels (Table 1). Those who continued to be on PD were significantly younger (*p* = 0.03), had lower systolic BP (*p* = 0.03), lower CRP (*p* = 0.01), NT-proBNP (*p* = 0.03), and CTRs (*p* < 0.01), than those who developed technique failure (Table 3). There was no significant difference with regard to the etiology of hemodialysis switch between patients with low and high NT-proBNP levels (Table 1). We found that PD patients with high NT-proBNP levels at baseline has significantly higher risk of technique failure compared to those with low levels (*p* = 0.01; Figure 3A). Cox proportional hazard regression analyses, incorporating the demographic profile, comorbidity, renal Kt/V, laboratory data, NT-proBNP, CTRs, and relative OH, showed that man (HR 1.91, *p* < 0.01, higher LnBNP levels (hazard ratio 1.13, *p* < 0.01), CTRs (HR 1.04, *p* < 0.01), and serum CRP (HR 1.01, *p* < 0.01) levels were independently predictive of an increased risk of technique failure among PD patients (Table 4).

Additionally, survivors were significantly younger (*p* < 0.01), had higher total Kt/V (*p* = 0.03), lower CRP (*p* = 0.03), and NT-proBNP (*p* = 0.01) levels than non-survivors (Table 3). We found that PD patients with high NT-proBNP levels at baseline has significantly higher risk of mortality compared to those with low levels (*p* = 0.03; Figure 3B). Cox regression analyses incorporating the demographic profile, comorbidity, Kt/V, laboratory data, LnBNP, CTRs, and relative OH, showed that mean (HR 3.05, *p* < 0.01), higher LnBNP levels (HR 1.56, *p* < 0.01), higher age (HR 1.1, *p* < 0.01), and CRP (HR 1.02, *p* < 0.01) levels were independently predictive of an increased risk of mortality among PD patients (Table 4).

## 4. Discussion

In the current study, we found that in a cohort of chronic PD patients, higher serum NT-proBNP levels at baseline were predictive of an increased risk of technique failure and overall mortality. This relationship was independent of other volume assessment results including the body composition parameters and CTRs, suggesting that NT-proBNP might influence the outcome, besides its role as a surrogate marker of body volume status.

The incidence of technique failure in our cohort was 0.08 episodes per patient-year (Table 3), which is within the range reported by others. Data from the Australia and New Zealand Dialysis and Transplant (ANZDATA) registry found that although the mean technique failure rate across institutes was 0.35 episodes per patient-year, the incidence between the centers could vary up to 7-fold [10]. A report from Japan suggested a comparatively lower rate of technique failure (0.16 episode per patient-year) [20], while another study from Taiwan similarly revealed a low rate of technique failure (0.08 episodes per patient-year) [21]. This might support the potential generalization of our results.

The role of NT-proBNP in patients undergoing long-term PD is somewhat controversial with respect to its pathophysiologic implications. An earlier study revealed that serum NT-proBNP levels did not correlate well with ECW volume in these patients, but were significantly correlated with left ventricular mass, ejection fraction, and cardiovascular mortality [22,23,24]. However, other studies revealed a significant relationship between NT-proBNP levels and extracellular water or body fluid status in PD patients [25,26]. Despite the inconsistency in the NT-proBNP/ECW relationship among long-term PD patients, results from most studies concur that NT-proBNP levels are closely associated with left ventricular dysfunction, and morbidity and mortality related to the heart in these patients. A repeat analysis of our data found that heart failure (ß = 0.97, *p* < 0.01) and the level of over-hydration (ß = 0.08, *p* < 0.01) were both independently associated with higher serum NT-proBNP levels in these patients, with a larger regression coefficient for heart failure. Consequently, we believe that the association between higher NT-proBNP levels and increased incidence of technique failure in this study can be better explained by the risk of cardiac dysfunction and related complications, than by an excess of body fluid in long-term PD patients. Indeed, a systematic review concluded that coronary artery disease and heart related ailments predispose PD patients to the risk of PD-related peritonitis [27], a major cause of technique failure, while those with cardiac morbidities are at a higher risk of decline in residual renal function and technique failure in the long run [10,28,29]. Furthermore, we found that the risk conferred by elevated NT-proBNP levels was independent of the over-hydration severity indicated by BCM readings (Table 4), neutralizing the effect of excess body fluid. Nonetheless, it can be difficult to completely unravel the complex relationship between cardiac dysfunction, excess body fluid, and technique failure; a study incorporating a larger sample size, or preferably a clinical trial design, is necessary to answer this question.

We also found that advanced age, male gender, and higher serum CRP levels were independent predictors of technique failure and mortality (Table 4). A study in Chinese PD patients showed that male gender increased the risk of PD peritonitis and possibly technique failure [30], while registry reports showed that higher age was a major risk factor for technique failure in PD patients [8,10]. Serum CRP measurement also predicts a risk of peritonitis-related complications and technique failure among PD patients [31]. It is plausible that PD patients of higher age tend to have visual impairment or cognitive disturbance, raising their risk of developing PD peritonitis, while local and systemic inflammation in PD patients might also contribute to cardiovascular risk and impaired immunologic response to pathogens, potentially leading to a higher risk of technique failure [32]. Finally, we also observed that serum PTH tended to be elevated in PD patients who survived compared to those who died (Table 3). This can be reasonable since mineral bone disorder, including renal osteodystrophy and vascular calcification may adversely influence the mortality of ESRD patients [33,34].

This study has its strengths, but they should be balanced against its limitations. The relationship between serum NT-proBNP and the risk of technique failure in PD patients has not been specifically addressed before, and the extent of study variables collected, ranging from clinical and laboratory parameters, to PD-specific parameters and bio-impedance assessment results, strongly increase the robustness of our findings. The follow-up duration is also adequate, with sufficient time interval to permit statistical comparison. However, the modest sample size and the single center nature of our cohort might limit the generalization of results to some extent [35]. We only measured serum albumin in these patients but not pre-albumin. In addition, previous studies suggested that a person-to-person variation of NT-proBNP levels in chronic PD patients could be high, and we could not confirm whether the biologic or analytic variations in NT-proBNP measurement could influence our findings [36]. Nonetheless, the within-person variation tends to be low (~20%), which suggests that a one-time measurement of NT-proBNP in a given individual might be reflective of his/her true average NT-proBNP levels. Although NT-proBNP levels are closely associated with cardiac function, we did not arrange echocardiography for our participants, and left ventricular ejection fraction data were unavailable. It is also likely that changes in comorbid illnesses and NT-proBNP levels over follow-up might influence our findings. Time-averaged NT-proBNP levels or repeatedly measured levels over a period of time will be necessary to better account for this issue.

## 5. Conclusions

In the current study, we sought to examine the predictability of baseline serum NT-proBNP levels for the subsequent risk of technique failure and mortality in a cohort of PD patients with a longitudinal follow-up for 3.6 years. We found that higher NT-proBNP levels were associated with a higher risk of technique failure and mortality in these patients, independent of demographic parameters, comorbidities, and several fluid status parameters including bio-impedance based over-hydration and image-based cardiothoracic ratios. Regular monitoring of NT-proBNP levels among PD patients may be useful for providing care for these patients.

## Figures and Tables

**Table 1:** Comparison of clinical characteristics between PD patients with low and high BNP levels.

	Total (*n* = 258)	Low BNP (*n* = 129)	High BNP (*n* = 129)	*p*-Value
Demographic profile				
Age	54 ± 12	54 ± 13	53 ± 12	0.33
Gender (F/M)	138/120	63/66	75/54	0.14
PD vintage (months)	42 ± 40	33 ± 36	50 ± 42	<0.01
BMI (kg/m^2^)	23.6 ± 3.5	23.8 ± 3.5	23.4 ± 3.6	0.39
Cause of ESRD				0.75
DMN	47 (18)	22 (17)	25 (19)	
CGN	150 (58)	79 (61)	71 (55)	
HTN	20 (8)	10 (8)	10 (8)	
Others	41 (16)	18 (14)	23 (18)	
Comorbidity				
Diabetes mellitus	63 (24)	33 (26)	30 (23)	0.98
Hypertension	217 (84)	106 (82)	111 (86)	0.87
Heart failure	26 (10)	4 (3)	22 (17)	<0.01
Coronary artery disease	32 (12)	14 (11)	18 (14)	0.9
Cirrhosis	2 (1)	0 (0)	2 (2)	0.57
Cancer (any)	15 (6)	6 (5)	9 (7)	0.89
Medications				
Diuretics	28 (11)	12 (9)	16 (12)	0.89
Statin	56 (22)	30 (23)	26 (20)	0.95
Beta-blocker	126 (49)	53 (41)	73 (57)	0.1
ACEI/ARB	103 (40)	45 (35)	58 (45)	0.43
Physical examination				
BP systolic (mmHg)	141 ± 22	139 ± 19	144 ± 25	0.11
BP diastolic (mmHg)	83 ± 13	83 ± 12	82 ± 15	0.86
PD variables				
PET results				
Low	10 (4)	4 (3)	6 (5)	0.94
Low Average	95 (37)	52 (40)	43 (33)	0.72
High Average	127 (49)	61 (47)	66 (51)	0.94
High	26 (10)	12 (9)	14 (11)	0.98
Fourth hour Glucose D/D0	0.37 ± 0.08	0.37 ± 0.08	0.36 ± 0.08	0.43
Fourth hour Creatinine D/P	0.68 ± 0.11	0.68 ± 0.11	0.69 ± 0.11	0.44
Peritoneal Kt/V	1.77 ± 0.37	1.68 ± 0.4	1.86 ± 0.3	<0.01
Renal Kt/V	0.23 ± 0.33	0.33 ± 0.37	0.13 ± 0.26	<0.01
Total Kt/V	2 ± 0.22	2.01 ± 0.24	2 ± 0.21	0.68
nPNA	1 ± 0.2	1.01 ± 0.18	0.99 ± 0.22	0.45
Laboratory profiles				
Hb (g/dL)	10 ± 1.5	10.3 ± 1.2	9.7 ± 1.7	<0.01
Albumin (gm/dL)	3.9 ± 0.4	3.9 ± 0.3	3.8 ± 0.4	0.13
BUN (mg/dL)	67 ± 16	69 ± 16	65 ± 17	0.06
Creatinine (mg/dL)	12.2 ± 2.8	12.3 ± 2.9	12.1 ± 2.7	0.74
PTH (pg/mL)	457 ± 423	472 ± 478	443 ± 362	0.57
CRP (mg/dL)	0.69 ± 1.72	0.66 ± 2	0.72 ± 1.39	0.77
NT pro-BNP (ng/mL)	582 ± 1216	23 ± 14	1141 ± 1530	<0.01
Body composition parameters				
Cardiac/thoracic ratio (%)	49 ± 7	48 ± 6	50 ± 7	<0.01
Relative OH (%)	10.3 ± 9.4	7.3 ± 8.7	13.3 ± 9.2	<0.01
ECW (L)	14.6 ± 3.2	14.6 ± 3.3	14.6 ± 3.1	0.87
ICW (L)	15.5 ± 3.5	16.1 ± 3.8	15 ± 3.2	<0.01
ECW/ICW	1 ± 0.1	0.9 ± 0.1	1 ± 0.1	<0.01
Follow-up duration (months)	43 ± 24	43 ± 22	44 ± 25	0.91
PD peritonitis incidence (per 100 months)	1.73	1.66	1.8	0.25
Switch to HD (%)				
PD peritonitis	46 (18)	23 (18)	23 (18)	1
Abdominal surgery	18 (7)	8 (6)	10 (8)	0.97
Catheter dysfunction	15 (6)	2 (2)	13 (10)	0.04
UF failure	3 (1)	1 (1)	2 (2)	0.95
Others	5 (2)	2 (2)	3 (2)	0.98

ACEI, angiotensin converting enzyme inhibitor; ARB, angiotensin receptor blocker; BUN, blood urea nitrogen; CGN, chronic glomerulonephritis; CRP, C-reactive protein; DMN, diabetic nephropathy; ECW, extracellular water; ESRD, end-stage renal disease; Hb, hemoglobin; HD, hemodialysis; HTN, hypertension; ICW, intracellular water; nPNA, normalized protein equivalent of total nitrogen appearance; NT pro-BNP, N-terminal pro-brain natriuretic peptide; OH, over-hydration; PD, peritoneal dialysis; PET, peritoneal equilibration test; PTH, parathyroid hormone; UF, ultrafiltration.

**Table 2:** Correlations between NT-proBNP levels and other clinical characteristics.

	Ln(NT-proBNP)	*p*-Value
Age	−0.1	0.11
Gender	0.23	<0.01
PD vintage	−0.05	0.47
Renal Kt/V	−0.33	<0.01
Hb (g/dL)	−0.19	<0.01
Albumin (gm/dL)	−0.1	0.11
Cardiac/thoracic ratio	0.21	<0.01
CRP (mg/dL)	0.04	0.54
Rel. OH (%)	0.43	<0.01
BP systolic (mmHg)	0.13	0.046
BP diastolic (mmHg)	0.03	0.63
ECW/ICW	0.35	<0.01

BP, blood pressure; CRP, C-reactive protein; ECW, extracellular water; Hb, hemoglobin; ICW, intracellular water; NT-proBNP, N-terminal pro-brain natriuretic peptide; Rel. OH, relative over-hydration; PD, peritoneal dialysis.

**Table 3:** Comparison of clinical characteristics between those with and without outcomes.

Variable	Remaining on PD (*n* = 130)	Technique Failure (*n* = 128)	*p*-Value	Survivors (*n* = 218)	Non-Survivors (*n* = 40)	*p*-Value
Age	52 ± 11	55 ± 13	0.03	52 ± 12	61 ± 11	<0.01
Gender (F/M)	76/54	62/66	0.46	123/95	15/25	0.18
PD vintage (months)	41 ± 43	43 ± 37	0.62	42 ± 39	43 ± 45	0.81
BMI (kg/m^2^)	23.3 ± 3.6	23.9 ± 3.4	0.15	23.5 ± 3.5	23.9 ± 3.5	0.56
Etiology of ESRD (%)						
DMN	19 (15)	28 (22)	0.52	34 (16)	13 (33)	0.09
CGN	86 (66)	64 (50)	0.08	132 (61)	18 (45)	0.34
HTN	7 (5)	13 (10)	0.56	16 (7)	4 (10)	0.95
Others	18 (14)	23 (18)	0.85	36 (17)	5 (13)	0.94
Diabetes mellitus	28 (22)	35 (27)	0.76	47 (22)	16 (40)	0.1
Hypertension	108 (83)	109 (85)	0.98	184 (84)	33 (83)	0.99
Heart failure	9 (7)	17 (13)	0.41	22 (11)	4 (10)	1.00
Coronary artery disease	13 (10)	19 (15)	0.71	24 (11)	8 (20)	0.47
Cirrhosis	1 (1)	1 (1)	1.00	2 (1)	0 (0)	0.95
Cancer (any)	4 (3)	11 (9)	0.31	9 (4)	6 (15)	0.06
Diuretics use	11 (8)	17 (13)	0.67	22 (10)	6 (15)	0.84
Statin use	29 (22)	27 (21)	0.99	48 (22)	8 (20)	0.99
Beta-blocker use	59 (45)	67 (52)	0.74	109 (50)	17 (43)	0.86
ACEI/ARB use	56 (43)	47 (37)	0.78	91 (42)	12 (30)	0.58
BP systolic (mmHg)	138 ± 22	144 ± 22	0.03	142 ± 23	139 ± 20	0.43
BP diastolic (mmHg)	82 ± 13	84 ± 14	0.37	83 ± 14	78 ± 12	0.03
Fourth hour Glucose D/D0	0.37 ± 0.07	0.37 ± 0.08	0.74	0.37 ± 0.08	0.38 ± 0.07	0.41
Fourth hour Creatinine D/P	0.69 ± 0.1	0.68 ± 0.11	0.89	0.69 ± 0.11	0.67 ± 0.09	0.39
Peritoneal Kt/V	1.76 ± 0.39	1.78 ± 0.35	0.76	1.78 ± 0.37	1.71 ± 0.35	0.3
Renal Kt/V	0.25 ± 0.34	0.22 ± 0.32	0.39	0.24 ± 0.33	0.22 ± 0.36	0.83
Total Kt/V	2.01 ± 0.22	1.99 ± 0.23	0.4	2.02 ± 0.23	1.93 ± 0.21	0.03
nPNA	0.99 ± 0.19	1.01 ± 0.22	0.42	1 ± 0.19	1.03 ± 0.26	0.44
Hb (g/dL)	10 ± 1.4	9.9 ± 1.6	0.56	10 ± 1.5	9.8 ± 1.2	0.37
Albumin (g/dL)	3.9 ± 0.3	3.9 ± 0.4	0.64	3.9 ± 0.4	3.8 ± 0.5	0.34
BUN (mg/dL)	67 ± 15	67 ± 18	0.97	67 ± 16	67 ± 18	0.8
Creatinine (mg/dL)	12.3 ± 2.9	12 ± 2.7	0.37	12.2 ± 2.7	12 ± 3.2	0.69
PTH (pg/mL)	488 ± 465	426 ± 375	0.24	479 ± 430	339 ± 369	0.05
CRP (mg/dL)	0.4 ± 1.1	1 ± 2.2	0.01	0.5 ± 1.12	1.7 ± 3.4	0.03
NT pro-BNP (ng/mL)	415 ± 1017	751 ± 1373	0.03	467 ± 1064	1206 ± 1724	0.01
Cardiac/thoracic ratio (%)	48 ± 6	50 ± 7	<0.01	49 ± 7	51 ± 7	0.05
Relative OH (%)	9.7 ± 9.1	11 ± 9.7	0.27	10.2 ± 9.2	11 ± 10.5	0.62
ECW (L)	14.4 ± 3.3	14.9 ± 3.1	0.22	14.6 ± 3.2	14.8 ± 3.4	0.62
ICW (L)	15.6 ± 3.7	15.5 ± 3.4	0.96	15.6 ± 3.5	15.1 ± 3.9	0.35
ECW/ICW	0.9 ± 0.1	1 ± 0.1	0.02	0.9 ± 0.1	1 ± 0.1	<0.01
Follow-up duration	50 ± 24	37 ± 21	<0.01	45 ± 24	36 ± 19	0.01

ACEI, angiotensin converting enzyme inhibitor; ARB, angiotensin receptor blocker; BUN, blood urea nitrogen; CGN, chronic glomerulonephritis; CRP, C-reactive protein; DMN, diabetic nephropathy; ECW, extracellular water; ESRD, end-stage renal disease; Hb, hemoglobin; HTN, hypertension; ICW, intracellular water; nPNA, normalized protein equivalent of total nitrogen appearance; NT pro-BNP, N-terminal pro-brain natriuretic peptide; OH, over-hydration; PD, peritoneal dialysis; PTH, parathyroid hormone.

**Table 4:** Cox proportional hazard regression analyses with technique failure and mortality as the dependent variables.

**Technique Failure**			
Variable	B ± SE	HR	*p*-Value
Age	0.01 ± 0.01	1.01	0.12
Male (vs. female)	0.65 ± 0.2	1.91	<0.01
Diabetes mellitus	−0.07 ± 0.24	0.94	0.54
Ln (NT-proBNP)	0.13 ± 0.05	1.13	<0.01
CT ratio (%)	0.04 ± 0.01	1.04	<0.01
CRP (mg/dL)	0.01 ± 0.004	1.01	<0.01
**Mortality**			
Variable	B ± SE	HR	*p*-Value
Age	0.09 ± 0.02	1.1	<0.01
Male (vs. female)	1.12 ± 0.38	3.05	<0.01
Diabetes mellitus	0.02 ± 0.39	1.02	0.95
CRP (mg/dL)	0.02 ± 0.004	1.02	<0.01
Ln(NT-proBNP)	0.44 ± 0.1	1.56	<0.01

CRP, C-reactive protein; CT, cardiothoracic; HR, hazard ratio; NT-proBNP, N-terminal pro-brain natriuretic peptide; SE, standard error.

## References

[1] Beddhu S., Greene T., Boucher R., Cushman W.C., Wei G., Stoddard G., Ix J.H., Chonchol M., Kramer H., Cheung A.K. (2018). Intensive systolic blood pressure control and incident chronic kidney disease in people with and without diabetes mellitus: Secondary analyses of two randomised controlled trials. Lancet Diabetes Endocrinol..

[2] Chao C.-T., Wang J., Wu H.-Y., Huang J.-W., Chien K.-L. (2018). Age modifies the risk factor profiles for acute kidney injury among recently diagnosed type 2 diabetic patients: A population-based study. GeroScience.

[3] Chao C.-T., Wang J., Chien K.-L. (2018). Both pre-frailty and frailty increase healthcare utilization and adverse health outcomes in patients with type 2 diabetes mellitus. Cardiovasc. Diabetol..

[4] Chaudhary K., Sangha H., Khanna R. (2011). Peritoneal Dialysis First: Rationale. Clin. J. Am. Soc. Nephrol..

[5] Vonesh E.F., Snyder J.J., Foley R.N., Collins A.J. (2006). Mortality studies comparing peritoneal dialysis and hemodialysis: What do they tell us?. Kidney. Int..

[6] Li P.K.-T., Chow K.M., Van de Luijtgaarden M.W., Johnson D.W., Jager K.J., Mehrotra R., Naicker S., Pecoits-Filho R., Yu X.Q., Lameire N. (2016). Changes in the worldwide epidemiology of peritoneal dialysis. Nat. Rev. Nephrol..

[7] Shih Y.-C., Guo A., Just P.M., Mujais S. (2005). Impact of initial dialysis modality and modality switches on Medicare expenditures of end-stage renal disease patients. Kidney Int..

[8] See E.J., Johnson D.W., Hawley C.M., Pascoe E.M., Badve S.V., Boudville N., Clayton P.A., Sud K., Polkinghorne K.R., Borlace M. (2018). Risk Predictors and Causes of Technique Failure within the First Year of Peritoneal Dialysis: An Australia and New Zealand Dialysis and Transplant Registry (ANZDATA) Study. Am. J. Kidney Dis..

[9] Nadeau-Fredette A.-C., Johnson D.W., Hawley C.M., Pascoe E.M., Cho Y., Clayton P.A., Borlace M., Badve S.V., Sud K., Boudville N. (2016). Center-Specific Factors Associated with Peritonitis Risk—A Multi-Center Registry Analysis. Perit. Dial. Int..

[10] Htay H., Cho Y., Pascoe E.M., Darssan D., Nadeau-Fredette A.C., Hawley C., Clayton P.A., Borlace M., Badve S.V., Sud K. (2017). Multicenter Registry Analysis of Center Characteristics Associated with Technique Failure in Patients on Incident Peritoneal Dialysis. Clin. J. Am. Soc. Nephrol..

[11] Wu H.-Y., Hung K.-Y., Huang T.-M., Hu F.-C., Peng Y.-S., Huang J.-W., Lin S.-L., Chen Y.-M., Chu T.-S., Tsai T.-J. (2012). Safety Issues of Long-Term Glucose Load in Patients on Peritoneal Dialysis—A 7-Year Cohort Study. PLoS ONE.

[12] Kim H.J., Lee J., Park M., Kim Y., Lee H., Kim D.K., Joo K.W., Kim Y.S., Cho E.J., Ahn C. (2017). Lower Education Level Is a Risk Factor for Peritonitis and Technique Failure but Not a Risk for Overall Mortality in Peritoneal Dialysis under Comprehensive Training System. PLoS ONE.

[13] Tian J.-P., Wang H., Du F.-H., Wang T. (2016). The standard deviation of extracellular water/intracellular water is associated with all-cause mortality and technique failure in peritoneal dialysis patients. Int. Urol. Nephrol..

[14] Paniagua R., Ventura M.D., Ávila-Díaz M., Hinojosa-Heredia H., Mendez-Duran A., Cueto-Manzano A., Cisneros A., Ramos A., Madonia-Juseino C., Belio-Caro F. (2010). NT-proBNP, fluid volume overload and dialysis modality are independent predictors of mortality in ESRD patients. Nephrol. Dial. Transplant..

[15] Kocyigit I., Sipahioglu M.H., Orscelik O., Unal A., Celik A., Abbas S.R., Zhu F., Tokgoz B., Dogan A., Oymak O. (2014). The Association between Arterial Stiffness and Fluid Status in Peritoneal Dialysis Patients. Perit. Dial. Int..

[16] Oh H.J., Lee M.J., Kwon Y.E., Park K.S., Park J.T., Han S.H., Yoo T.H., Kim Y.L., Yang C.W., Kim N.H. (2015). Which Biomarker is the Best for Predicting Mortality in Incident Peritoneal Dialysis Patients: NT-ProBNP, Cardiac TnT, or hsCRP? A Prospective Observational Study. Medicine.

[17] Caetano C., Valente A., Oliveira T., Garagarza C. (2016). Body Composition and Mortality Predictors in Hemodialysis Patients. J. Ren. Nutr..

[18] Chao C.-T., Chan D.-C., Huang J.-W. (2017). Frail Phenotype Might Be Associated With Higher Appendicular but Not Truncal Fat among End-Stage Renal Disease Patients. J. Pain Symptom Manag..

[19] Shen J.I., Mitani A.A., Saxena A.B., Goldstein B.A., Winkelmayer W.C. (2013). Determinants of Peritoneal Dialysis Technique Failure in Incident US Patients. Perit. Dial. Int..

[20] Matsui M., Akai Y., Samejima K.I., Tsushima H., Tanabe K., Morimoto K., Tagawa M., Saito Y. (2017). Prognostic Value of Predialysis Indices for Technique Failure and Mortality in Peritoneal Dialysis Patients. Ther. Apher. Dial..

[21] Hsieh Y.-P., Chang C.-C., Kor C.-T., Yang Y., Wen Y.K., Chiu P.F., Lin C.C. (2017). Relationship between uric acid and technique failure in patients on continuous ambulatory peritoneal dialysis: A long-term observational cohort study. BMJ Open.

[22] Lee J.-A., Kim D.-H., Yoo S.-J., Oh D.J., Yu S.H., Kang E.T. (2006). Association between serum N-terminal pro-brain natriuretic peptide concentration and left ventricular dysfunction and extracellular water in continuous ambulatory peritoneal dialysis patients. Perit. Dial. Int..

[23] Wang A.Y., Lam C.W., Yu C.M., Wang M., Chan I.H., Zhang Y., Lui S.F., Sanderson J.E. (2007). N-Terminal Pro-Brain Natriuretic Peptide: An Independent Risk Predictor of Cardiovascular Congestion, Mortality, and Adverse Cardiovascular Outcomes in Chronic Peritoneal Dialysis Patients. J. Am. Soc. Nephrol..

[24] Paniagua R., Amato D., Mujais S., Vonesh E., Ramos A., Correa-Rotter R., Horl W.H. (2008). Predictive Value of Brain Natriuretic Peptides in Patients on Peritoneal Dialysis: Results from the ADEMEX Trial. Clin. J. Am. Soc. Nephrol..

[25] Chung J.H., Yun N.R., Ahn C.Y., Lee W.S., Kim H.L. (2008). Relationship between Serum N-Terminal Pro-Brain Natriuretic Peptide Level and Left Ventricular Dysfunction and Extracellular Water in Continuous Ambulatory Peritoneal Dialysis Patients. Electrolyte Blood Press..

[26] Papakrivopoulou E., Lillywhite S., Davenport A. (2012). Is N-terminal probrain-type natriuretic peptide a clinically useful biomarker of volume overload in peritoneal dialysis patients?. Nephrol. Dial. Transplant..

[27] Kerschbaum J., Konig P., Rudnicki M. (2012). Risk Factors Associated with Peritoneal-Dialysis-Related Peritonitis. Int. J. Nephrol..

[28] Liao C.-T., Shiao C.-C., Huang J.-W., Hung K.Y., Chuang H.F., Chen Y.M., Wu K.D., Tsai T.J. (2008). Predictors of faster decline of residual renal function in Taiwanese peritoneal dialysis patients. Perit. Dial. Int..

[29] Liao C.-T., Chen Y.-M., Shiao C.-C., Hu F.-C., Huang J.-W., Kao T.-W., Chuang H.-F., Hung K.-Y., Wu K.-D., Tsai T.-J. (2009). Rate of decline of residual renal function is associated with all-cause mortality and technique failure in patients on long-term peritoneal dialysis. Nephrol. Dial. Transplant..

[30] Tian Y., Xie X., Xiang S., Yang X., Lin J., Zhang X., Shou Z., Chen J. (2017). Risk Factors and Outcomes of Early-Onset Peritonitis in Chinese Peritoneal Dialysis Patients. Kidney Blood Press. Res..

[31] Moon S.J., Han S.H., Kim D.K., Lee J.E., Kim B.S., Kang S.W., Choi K.H., Lee H.Y., Han D.S. (2008). Risk factors for adverse outcomes after peritonitis-related technique failure. Perit. Dial. Int..

[32] Li P.K., Ng J.K., McIntyre C.W. (2017). Inflammation and Peritoneal Dialysis. Semin. Nephrol..

[33] Hruska K.A., Seifert M., Sugatani T. (2015). Pathophysiology of the chronic kidney disease-mineral bone disorder. Curr. Opin. Nephrol. Hypertens..

[34] Chao C.-T., Liu Y.-P., Su S.-F., Yeh H.-Y., Chen H.-Y., Lee P.-J., Chen W.-J., Lee Y.-M., Huang J.-W., Chiang C.-K. (2017). Circulating microRNA-125b predicts the presence and progression of uremic vascular calcification. Arterioscler. Thromb. Vasc. Biol..

[35] Chao C.-T., Hsu Y.-H., Chang P.-Y., He Y.-T., Ueng R.-S., Lai C.-F., Chiang C.-K., Huang J.-W., Huang S.-J. (2015). Simple self-report FRAIL scale might be more closely associated with dialysis complications than other frailty screening instruments in rural chronic dialysis patients. Nephrology.

[36] Fahim M.A., Hayen A., Horvath A.R., Dimeski G., Coburn A., Johnson D.W., Hawley C.M., Campbell S.B., Craig J.C. (2015). N-Terminal Pro–B-Type Natriuretic Peptide Variability in Stable Dialysis Patients. Clin. J. Am. Soc. Nephrol..

